# A new vector for efficient gene targeting to the *pyrG* locus in *Aspergillus niger*

**DOI:** 10.1186/s40694-015-0012-4

**Published:** 2015-03-14

**Authors:** Mark Arentshorst, Ellen L Lagendijk, Arthur FJ Ram

**Affiliations:** Molecular Microbiology and Biotechnology, Institute of Biology Leiden, Leiden University, Sylviusweg 72, 2333 BE Leiden, The Netherlands

**Keywords:** Reporter gene, Luciferase activity, Promoter analysis

## Abstract

**Background:**

The possibility for efficient gene targeting for the controlled integration of DNA constructs is an important tool in fungal genetics.

**Findings:**

In this study, we report a new targeting vector based on the *pyrG* marker in *Aspergillus niger*. The DNA sequence to be targeted is surrounded by two fragments of the *pyrG* gene to allow homologous recombination of the recombinant DNA at the *pyrG* locus. The 5’ end of the targeting cassette contains a non-functional truncated *pyrG* open reading frame (first 112 bases deleted) and the 3’ untranslated region (3’ UTR). At the 3’ end, the targeting cassette consists of the 3’ flanking region of the *pyrG* gene. A unique *Not*I site between the flanks allows the insertion of a gene of interest. The linearized targeting cassette is transformed to the *A. niger pyrG* mutant strain AB4.1 or a derivative thereof. By using a constitutively expressed luciferase reporter gene (*mluc*) as an example, it is shown that the targeting system is efficient as 4 out of 6 (67%) AB4.1 transformants and 51 out of 66 (77%) MA169.4 (*ku70* ^*−*^) transformants contained the reporter gene at the *pyrG* locus. A luciferase (lux) activity assay, performed with independently obtained transformants in which the *mluc* reporter was integrated at the *pyrG* locus, showed comparable and reproducible lux activities.

**Conclusion:**

The new *pyrG* targeting vector is an important improvement to the existing method for gene targeting in *A. niger.* Although the vector is specific for *A. niger,* the presented design and approach is easily applicable for constructing integration vectors for other fungi.

## Finding

**Figure 1:**
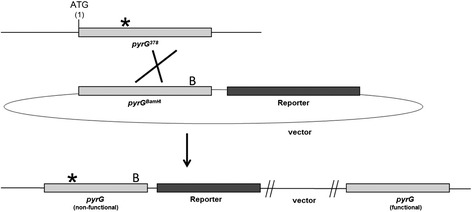
**Schematic representation of integration of a reporter construct after a single crossover event using the** pyrG* **targeting system.** This system was described previously by van Gorcom and van den Hondel [9]. Strain AB4.1 contains a base deletion at position 378 in the *pyrG* open reading frame indicated with a *. A circular plasmid containing the reporter gene and the mutated *pyrG* gene (*pyrG* ^*Bam*HI^) is transformed to AB4.1 and a single cross over at the *pyrG* locus leads to integration into the genome. Note that the entire vector is integrated in this system. The pyrG* fragment is a located on a 3.8 kb *Xba*I subclone and can be inserted in a vector for targeted integration.

The *pyrG* gene of *Aspergillus niger* (An12g03570) is homologous to the *Saccharomyces cerevisiae URA3* gene. In *A. niger,* as well as in other fungi, the *pyrG* marker is popular because it is a stringent auxotrophic marker which can be fully supplemented with uridine or uracil [1-3]. Another advantage of the *pyrG* marker over other auxotrophic markers is that it is counter selectable and *pyrG* mutants can be obtained by isolating 5-fluoro-orotic resistant mutants [4,5]. The *pyrG* mutant strain AB4.1 is often used in *A. niger* genetics [1], for instance in numerous studies to create gene deletion mutants using the *pyrG* gene of *A. oryzae* as a heterologous marker, initially developed by de Ruijter-Jacobs et al. [6]. The *A. niger Δku70, pyrG* ^*−*^ mutant MA70.15 [7] and *ku70* ^*−*^ *, pyrG* ^*−*^ mutant MA169.4 [8], are derived from AB4.1. Sequence analysis of the *pyrG* gene in the AB4.1 mutant showed a deletion of a cytosine at position 378 bp in the *pyrG* gene, causing an out of frame mutation after 103 amino acids. The AB4.1 mutant has been used to set up a system for targeted integration of e.g. reporter constructs to the *pyrG* locus [9]. This system, referred to as the pyrG* (*pyrG* star) system, makes use of the AB4.1 mutant, in combination with a plasmid based mutated *A. niger pyrG* gene. The mutation in the plasmid based *pyrG* gene was introduced by filling in the *Bam*HI restriction site (position 828 bp in the *pyrG* ORF) that is present in the protein-coding region of the gene, causing a frame shift. The vector containing the *pyrG* ^*Bam*HI^ will not be functional when ectopically integrated and will therefore not give rise to uridine prototrophic transformants. However, targeted integration of the *pyrG* ^*Bam*HI^ at the *pyrG* locus of strain AB4.1 via a single cross over will result in the integration of the vector at the *pyrG* locus (Figure 1). The pyrG* system has been successfully used in several studies for the targeted integration of reporters in gene expression studies [10,11], or for the controlled integration of expression cassettes [12] and GFP-fusion proteins [13].

However, there are certain drawbacks of using the pyrG* method. Transformation frequencies are on average 10 to 20 times lower compared to regular *pyrG* transformations, often resulting in only one or two transformants per transformation. In addition, the percentage of successful integration of the reporter construct, varying between 10 to 50%, is rather low, probably due to a recombination or repair event that restores the mutation in the *pyrG* gene in AB4.1. Another disadvantage of the pyrG* system is that after targeted integration, two *pyrG* repeats are present around the construct, making the loss of the reporter construct via a loop out event possible (Figure 1). Finally, despite the well established positive effect on gene targeting efficiencies in *ku70* mutants [7,8], we noticed that deletion of the *ku70* gene did not improve transformation or targeted integration frequencies of the pyrG* plasmid. The reason for this is not known and systematic studies to analyse homologous integration using circular (pyrG*) or linear DNA fragments in wild type or *ku70* strains to clarify this have not been reported. These drawbacks make the construction of *A. niger* strains with targeted integration, such as of reporter constructs, a time consuming and laborious exercise.

**Figure 2:**
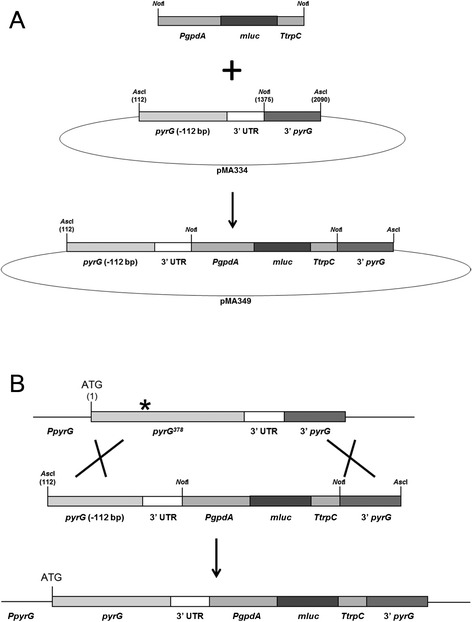
**Schematic representation of integration of a reporter construct after a double crossover event using the** pyrG** **targeting system. A)** The truncated *pyrG* gene (-112) + 3’ UTR fragment (1255 bp) was amplified by PCR using primers ABpyrGP12f and ABpyrGP10r. The 3’ *pyrG* fragment (684 bp) was amplified by PCR using primers ABpyrGP11f and ABpyrGP13r. Both PCR products were digested (*Eco*RI-*Not*I for *pyrG-*3’ UTR and *Not*I-*Sst*II for 3’ *pyrG*) and ligated in *Eco*RI-*Sst*II opened pBluescriptSK, yielding plasmid pMA334. The *mluc* reporter cassette was obtained by PCR using SL1 and TtrpCP2rev-NotI as primers and pMA313 (containing *PgpdA-mluc-TtrpC*-*AOpyrG,* unpublished vector) as template. pMA334 was opened with *Not*I and the *Not*I digested *Pgpd-mluc-TtrpC* fragment was inserted, giving plasmid pMA349. Both plasmids have been deposited at Fungal Genetic Stock Centre. pMA349 was digested with *Asc*I to release the complete pyrG** targeting transformation DNA. **B)** Integration of the pyrG** targeting construct via a double cross over at the *pyrG* locus.

In this study, a new *pyrG* targeting vector is presented to facilitate the construction of strains in which a reporter construct or another gene of interest is targeted to the *pyrG* locus, which is named pyrG** (*pyrG* double star). The targeting vector (pMA334) is schematically depicted in Figure 2A and consists of a 5’ truncated non-functional *pyrG* gene, the *pyrG* 3’ UTR, a unique *Not*I site, and a 3’ *pyrG* flanking region. The position of the *Not*I site has been chosen directly behind the end of the 3’ UTR of the *pyrG* mRNA and is based on RNA seq data (personal communication with P. Punt). The *Not*I site can be used to clone a particular DNA fragment (e.g. promoter reporter construct) in the targeting vector. Transformation of the linear DNA fragment will only result in a functional *pyrG* gene when the targeting vector integrates at the *pyrG* locus via a double cross over event (Figure 2B). The first cross over must occur between the 5’ end of the truncated *pyrG* gene on the plasmid and the *pyrG* mutation in AB4.1. The second cross over must occur at the 3’ flank for a complete integration of the cassette. In theory, a double crossover event could also occur between the 5’ truncated non-functional *pyrG* gene and the *pyrG* 3’ UTR, giving rise to *pyrG* prototrophic transformants that do not contain the reporter gene.

**Figure 3:**
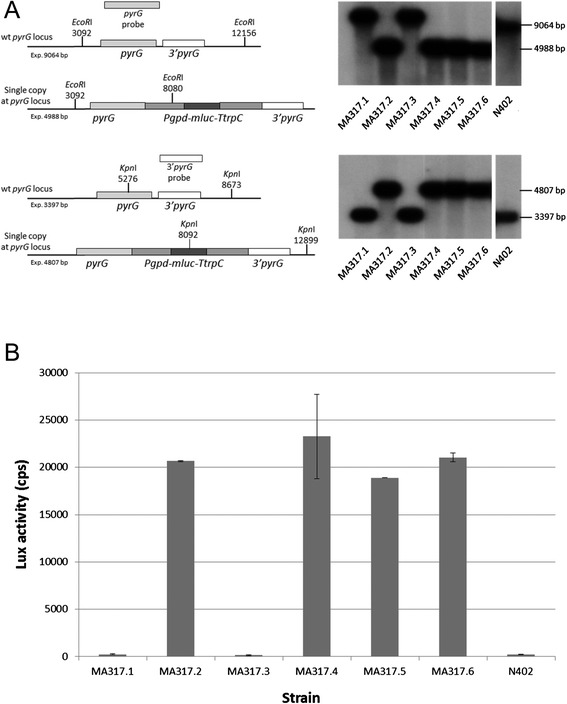
**Analysis of** ***A. niger*** **MA317 transformants containing the** ***Pgpd-mluc*** **reporter construct** using the pyrG** **targeting method. A)** Southern blot analysis. Genomic DNA was restricted with *Eco*RI or *Kpn*I and size fractioned by electrophoresis on a 1.0 % agarose gel. For hybridisation, ^32^P-labelled *pyrG* probe (1255 bp, Figure 2A) or 3’ *pyrG* probe (684 bp, Figure 2A) were used. When digested with *Eco*RI and using the *pyrG* probe (upper panel), a signal of 9.0 kb corresponds with the wild-type *pyrG* locus, while a signal of 4.9 kb corresponds with integration of the *Pgpd-mluc* cassette at the *pyrG* locus. When digested with *Kpn*I and using the 3’ *pyrG* probe (lower panel), a signal of 3.3 kb corresponds with the wild-type *pyrG* locus, while a signal of 4.8 kb corresponds with integration of the *Pgpd-mluc* cassette at the *pyrG* locus. Strains MA317.1 and MA317.3 have the wild-type *pyrG* locus, while strains MA317.2 and MA317.4-6 contain the *Pgpd-mluc* cassette at the *pyrG* locus. **B)** Lux activity assay. The lux activity assay described by Meyer et al. [12] has been slightly modified. Briefly, 100 μL of 2 x Minimal Medium [5] with 0.006 % yeast extract (w/v), 76 μL deionized water, 4 μL 25 mM luciferin (Promega, E1605) and 20 μL spore suspension (7.5*10^5^ spores/mL) was pipetted together (in triplicate) in a well of a white, clear bottom, 96 wells plate (Greiner Bio-one, ref 655095) and incubated for 24 hours at 30 °C in the EnSpire Multiplate Reader (Perkin) with continuous measuring of lux and OD. Lux activities after 16 h of incubation are shown here. Strains MA317.1 and MA317.3 have no lux activity, while strains MA317.2 and MA317.4-6 show comparable lux activities.

To test the efficiency of the pyrG** targeting system, the luciferase reporter gene *mluc* [12] was cloned behind the constitutive *gpdA* promoter and ligated into targeting vector pMA334, giving plasmid pMA349 (Figure 2A). Linear DNA was isolated by digestion of pMA349 using *Asc*I and after purification, 20 μg of DNA was transformed [5] into *A. niger* strain AB4.1 (*pyrG* ^*−*^) (for strains and primers used in this study, see Table 1 and Table 2 respectively), resulting in 6 primary transformants. To check if integration of the reporter construct on the *pyrG* locus has occurred, all transformants were purified and analysed by Southern blot (Figure 3A). The result of the Southern blot analysis shows that 4 out of 6 transformants contain the reporter construct at the *pyrG* locus, while the other 2 transformants have a wild-type *pyrG* locus. The luciferase activity of these transformants was determined in a lux activity assay (Figure 3B), and only the strains that contain the reporter construct show lux activity.

**Table 1:** **Strains used in this study**

**Strain**	**Genotype**	**Description**	**Reference**
N402	*cspA1*	derivative of N400	[14]
AB4.1	*cspA1, pyrG378*	UV mutant of N402	[1]
MA169.4	*cspA1, pyrG378, kusA::DR-amdS-DR*	*ku70* disruption in AB4.1	[8]
MA317.1-6	*cspA1, PgpdA-mluc-TtrpC on pyrG locus*	*PgpdA-mluc-TtrpC* on *pyrG* locus in AB4.1	This study
MA565.1-66	*cspA1, kusA::DR-amdS-DR, PgpdA-mluc-TtrpC on pyrG locus*	*PgpdA-mluc-TtrpC* on *pyrG* locus in MA169.4	This study

**Table 2:** **Primers used in this study**

**Primer name**	**Sequence 5’-3’**	**Used for**
ABpyrGP12for-EcoRI-AscI	CG*GAATTCGG CGCGCC*CGGC TGACGTTACC ACCACT*	*pyrG-*3’ UTR PCR (Figure 2A)
ABpyrGP10rev-NotI	AAGGAAAAAA *GCGGCCGC*AG TCAGACCTAA TGCCTCGGG	*pyrG-*3’ UTR PCR (Figure 2A)
ABpyrGP11for-NotI	AAGGAAAAAA *GCGGCCGC*CG TCGCGTGATA AGGGTTG	3’ *pyrG* PCR (Figure 2A)
ABpyrGP13rev-SacI-AscI	C*GAGCTCGGC GCGCC*TCGGG TCAATTTCCT CTGTTG	3’ *pyrG* PCR (Figure 2a)
SL1	ATTTGCGGCC GCAGAACGCC GAGAAGAACT GG	*PgpdA-mluc* PCR (Figure 2A)
TtrpCP2rev-NotI	AAGGAAAAAA *GCGGCCGC*TC TAGAAAGAAG GATTACCTC	*PgpdA-mluc* PCR (Figure 2A)

*Relevant restriction sites are shown in italic.

In the pyrG** system, the reporter construct has to integrate at the *pyrG* locus via homologous integration. In order to test the performance of the pyrG** system in a *ku70* ^−^background, strain MA169.4 (*pyrG* ^*−*^, *ku70* ^*−*^) was transformed with 10 μg of linear DNA, isolated by digestion of plasmid pMA349 with *Asc*I. In total 66 primary transformants were obtained and purified. These transformants were analysed for their luciferase activity in a lux assay (data not shown), resulting in 51 out of 66 (77%) strains that show lux activity. Southern blot analysis (data not shown) of 15 selected strains showed that 13 strains, which showed comparable lux activities in the lux assay, contain the lux reporter construct at the *pyrG* locus. In the other two strains, that were negative in the lux assay, the reporter construct was not present. No ectopic integrations were detected in these 15 transformants.

In the experiments described above, the results indicate that the pyrG** targeting system is both useful in wild-type and *ku70* ^*−*^ strains, even though the transformation frequency in the wild-type strain is much lower. It is likely that this lower frequency of transformation is due to the ectopic integration of the cassette, which does not result in transformants as this integration does not reconstitute the *pyrG* gene.

The new targeting system has recently been successfully used in two independent studies in our laboratory. In the first study (A-M Burggraaf-van Welzen, unpublished results) 4 different *ku70* ^*−*^ strains and 2 different reporter genes were used. Out of 28 transformants analysed, 23 transformants contained the reporter construct at the *pyrG* locus (82%). In the second study (J. Niu, unpublished results), a *Δku70* strain was transformed with 6 different reporter constructs. Out of 122 primary transformants analysed, 105 transformants contained the reporter construct (86%). Southern analysis of 24 transformants (four of each construct) confirmed integration at the *pyrG* locus in all transformants analysed. These studies further confirm the efficiency and ease at which transformants with targeted integrations are obtained.

The described method to obtain transformants with targeted integration is not restricted to *pyrG* mutants, but can also be used for other auxotrophic markers. Prerequisite is that the mutation in the auxotrophic marker is determined to allow design of the targeting cassette.

## Availability of supporting data

The data set supporting the results of this article is included within the article. Plasmids and plasmids maps and DNA sequences are deposited at Fungal Genetics Stock Centre.
